# TGF-β Increases MFGE8 Production in Myeloid-Derived Suppressor Cells to Promote B16F10 Melanoma Metastasis

**DOI:** 10.3390/biomedicines9080896

**Published:** 2021-07-26

**Authors:** Heejin Lim, Taewoo Yang, Wongeun Lee, Sung-Gyoo Park

**Affiliations:** 1 School of Life Sciences, Gwangju Institute of Science and Technology, Gwangju 61005, Korea; heejin425@gm.gist.ac.kr (H.L.); yangtw@gist.ac.kr (T.Y.); wodrl1234@gmail.com (W.L.); 2 Institute of Pharmaceutical Sciences, College of Pharmacy, Seoul National University, Seoul 08826, Korea

**Keywords:** myeloid-derived suppressor cells, tumor, metastasis, TGF-β, MFGE8

## Abstract

There is growing evidence that myeloid-derived suppressor cells (MDSCs) are directly involved in all stages leading to metastasis. Many mechanisms for this effect have been proposed, but mechanisms of coregulation between tumor cells and MDSCs remain poorly understood. In this study, we demonstrate that MDSCs are a source of milk fat globule-epidermal growth factor (EGF) factor 8 (MFGE8), which is known to be involved in tumor metastasis. Interestingly, TGF-β, an abundant cytokine in the tumor microenvironment (TME), increased MFGE8 production by MDSCs. In addition, co-culturing MDSCs with B16F10 melanoma cells increased B16F10 cell migration, while MFGE8 neutralization decreased their migration. Taken together, these findings suggest that MFGE8 is an important effector molecule through which MDSCs promote tumor metastasis, and the TME positively regulates MFGE8 production by MDSCs through TGF-β.

## 1. Introduction

Myeloid-derived suppressor cells (MDSCs) were first identified as immune-suppressive myeloid cells in the 1970s [1]. Since then, MDSCs have been studied as important immune regulators with pro-metastatic [2,3,4,5] and anti-inflammatory properties [6,7]. MDSCs are a significant component of the tumor microenvironment (TME) [2,8] and are considered a biomarker of tumor progression [9]. Previous studies have demonstrated a positive correlation between various tumors and MDSCs [8,10,11]. For example, tumor-derived factors affect MDSCs accumulation as well as their activation [12,13,14]. In turn, MDSCs secrete cytokines to suppress immune cells such as CD8^+^ T cells, which are involved in anti-tumor immune responses [15,16].

Metastasis is a complex process through which tumor cells spread to other organs. It progresses through several steps, including epithelial-mesenchymal transition (EMT), local invasion, neovascularization, and pre-metastatic niche formation [17,18,19,20]. MDSCs have been studied as an important regulator of tumor metastasis. For example, MDSCs promote tumor growth by remodeling the TME to facilitate angiogenesis [21,22]. MDSCs are also known to establish a pre-metastatic niche and induce EMT [19,20]. These data highlight that elucidating the mechanisms through which MDSCs communicate with tumor cells is important for understanding tumor progression and for the development of anti-tumor therapies.

Previously, we demonstrated the importance of TGF-β in the immunosuppressive function of MDSCs during inflammation [7]. However, TGF-β is also an important component of the TME and a mediator of tumor progression and immune modulation in cancer [23,24,25]. Thus, we considered the possibility that TGF-β-induced MDSCs activation could also affect the tumor-related response. Microarray analysis identified several potential targets, including milk fat globule-epidermal growth factor (EGF) factor 8 (MFGE8). As its name suggests, MFGE8 was first discovered as a component of milk fat globules and was known as an apoptosis mediator [26]. However, growing evidence suggested its role in tumor progression, EMT, chemoresistance, cancer stem cell activation [27,28,29,30], and anti-inflammation [31,32]. Given these properties, MFGE8 has been studied as an inducer of tumor metastasis, and tumor cells and myeloid cells such as tumor-associated macrophages (TAMs) are thought to be the main source of MFGE8 [27,28,29,33]. 

In this study, we identify MDSCs as a source of MFGE8, reinforcing the relationship between MDSCs and the TME. Furthermore, we hypothesized that MFGE8 could be an important effector molecule used by MDSCs to promote the metastasis of B16F10 melanoma. Our results show that MFGE8 is upregulated in MDSCs in response to TGF-β treatment. We also identified an interaction between MDSCs and B16F10 melanoma that increased EMT and melanoma cell invasion, showing that B16F10 metastasis was affected by MDSC-derived MFGE8. Together, these findings suggest that MDSC-derived MFGE8 could be a valuable target for cancer therapies.

## 2. Materials and Methods

### 2.1. Mice and Cell Culture

The mouse melanoma cell line B16F10 was purchased from the Korean Cell Line Bank (KCLB, Seoul, Korea). B16F10 cells were cultured in Dulbecco’s modified Eagle medium (DMEM; Hyclone, Marlborough, MA, USA) supplemented with 10% (*v*/*v*) fetal bovine serum (FBS; Hyclone), 100 U/mL penicillin, and 100 µg/mL streptomycin (Gibco). In some experiments, cells were treated with 10 ng/mL of recombinant mouse MFGE8 protein (short isoform, Novus, Centennial, CO, USA).

Mouse bone marrow was harvested from the femurs and tibias of 6–10-week-old female C57BL/6 mice. For in vitro differentiation, bone marrow cells were cultured in Rosewell Park Memorial Institute (RPMI) 1640 medium (Hyclone) supplemented with 10% fetal bovine serum, 100 U/mL penicillin, 100 µg/mL streptomycin, recombinant murine IL-4 (10 ng/mL, Peprotech, Cranbury, NJ, USA), recombinant murine GM-CSF (10 ng/mL, Peprotech), 2-mercaptoethanol (50 μM, Sigma-Aldrich, St. Louis, MO, USA), and recombinant mouse TGF-β1 (1 ng/mL, R&D Systems, Minneapolis, MN, USA). All experiments using mice were approved by the Institutional Animal Care and Use Committee of Gwangju Institute of Science and Technology (GIST-2020-086, approved on 26 August 2020).

### 2.2. FACS and Antibodies

In vitro-differentiated MDSCs were sorted using a BD FACSAria III (BD Bioscience, San Jose, CA, USA). Cells were stained with monoclonal antibodies against CD11b (M1/70, ebioscience, San Diego, CA, USA), Ly6G/Ly6C (RB6/8C5, ebioscience), Ly6G (1A8, BD Pharmingen, San Diego, CA, USA), and Ly6C (HK1.4, ebioscience). CD11b^+^Gr-1^+^Ly6G^high^Ly6C^low^ cells were sorted as granulocytic MDSCs (Gr-MDSCs), and CD11b^+^Gr-1^+^Ly6G^low^Ly6C^high^ cells were sorted as monocytic MDSCs (Mo-MDSCs). 

### 2.3. Retroviral Overexpression

To overexpress *Mfge8*, retrovirus was generated from Phoenix Eco cells by transient transfection with a retroviral pMigR1_GFP vector. Bone marrow cells were seeded for 24 h, then proliferating bone marrow cells were treated with viral supernatant containing polybrene (hexadimethrine bromide; Sigma-Aldrich) and centrifuged. GFP-positive cells were sorted by flow cytometry.

### 2.4. RNA Extraction and Quantitative RT-PCR

Total RNA from Mo- and Gr-MDSCs was isolated using the RNeasy Micro Kit (Qiagen, Hilden, Germany) and total RNA from B16F10 cells was isolated using TRI Reagent (Molecular Research Center, Cincinnati, OH, USA). Using the extracted RNA, cDNA was synthesized with TOPscript RT DryMIX (enzynomics, Daejeon, Korea), and relative mRNA levels were determined by quantitative PCR using TOPreal qPCR 2X PreMix (enzynomics) and the CFX Connect Real-Time System (Bio-Rad, Hercules, CA, USA). The following primer pairs were used: *GAPDH* (Forward, 5′-GCC TTC TCC ATG GTG GTG AA; Reverse, 5′-GCA CAG TCA AGG CCG AGA AT), *Mfge8* (Forward, 5′-CAA GGA ATG GCT GCA GGT TGA C; Reverse, 5′-AAG TTG CCC TGG AAG ACC TTG C), *Arginase1* (Forward, 5′-TCC ACC CTG ACC TAT GTG TCA TTT; Reverse, 5′-CGT CTC GCA AGC CAA TGT ACA); *iNOS* (Forward, 5′-TTC ACC CAG TTG TGC ATC GAC CTA; Reverse, 5′-TCC ATG GTC ACC TCC AAC ACA AGA), *IL-10* (Forward, 5′-GCA CTG CTA TGC TGC CTG CTC TTA CTG A; Reverse, 5′-AGC TTC TCA CCC AGG GAA TTC AAA TGC T), *TGF-β* (Forward, 5′-CTC CCG TGG CTT CTA GTG C; Reverse, 5′-GCC TTA GTT TGG ACA GGA TCT G); *E-cadherin* (Forward, 5′-ACA CGT TCA TGG ATC AGA AGA TCA C; Reverse, 5′-ACA GGA CCA GGA GAA GAG TGC C), *Vimentin* (Forward, 5′-CCG CTT TGC CAA CTA CAT; Reverse, 5′-GGA TTC CAC TTT CCG TTC); *Twist1* (Forward, 5′-AGC GGG TCA TGG CTA ACG; Reverse, 5′-GGA CCT GGT ACA GGA AGT CGA); and *MMP13* (Forward, 5′-GTG ACT GGC AAA CTT GAT GA; Reverse, 5′-TCA CAT CAG ACC AGA CCT TG). The relative mRNA expression was calculated by the ΔΔC_T_ method, with normalization to the expression of *GAPDH*.

### 2.5. Enzyme-Linked Immunosorbent Assay (ELISA)

After sorting of MDSCs, 1.25 × 10^5^ cells were seeded in 96-well plates for 24 h. Supernatant was harvested and used for measurement of MFGE8 by ELISA. The Mouse Milk Fat Globule 1 ELISA kit (Abcam, Cambridge, UK) was used according to the manufacturer’s protocol. The TGF-β ELISA was conducted using the Human/Mouse TGF β1 Uncoated ELISA kit (Invitrogen, Carlsbad, CA, USA) according to the manufacturer’s protocol with supernatant from B16F10 cells after 24 h of culture.

### 2.6. Water-Soluble Tetrazolium Salts (WST) Assay

1 × 10^4^ B16F10 cells were seeded in 96-well plates. After overnight culture, cells were treated with 10 ng/mL MFGE8. On Days 0, 1, 2, and 3, the optical density at 450 nm (OD450) was measured after 1 h of incubation with EZ Cytox (DoGenBio, Seoul, Korea).

### 2.7. Migration and Invasion Assays

For migration assays, 5 × 10^5^ B16F10 cells suspended in serum-free DMEM were loaded in the upper chamber of a Falcon Permeable Support for 24-well Plate with 8.0 µm Transparent PET Membrane. The lower part of the chamber was filled with DMEM containing 10% FBS and MFGE8 (10 ng/mL). After 24 h, non-migrating cells were removed with a cotton swab and migrating cells were fixed with 70% ethanol and stained with 0.5% crystal violet solution (BD Bioscience). 

For invasion assays, transwell inserts were coated with 50 μL Matrigel Matrix (Corning, NY, USA) mixed with DMEM (1:4 ratio). 5 × 10^5^ B16F10 cells were loaded in the upper chamber and MFGE8 (10 ng/mL) was added to the lower well. After 48 h, non-invading cells were removed with a cotton swab and invading cells were fixed with 70% ethanol and stained with 0.5% crystal violet solution.

For co-culture experiments, 3 × 10^5^ B16F10 cells stained with CellTrace Violet (Invitrogen) were loaded in the upper chamber, while 3 × 10^5^ CD11b^+^Gr-1^+^ MDSCs were loaded in the lower chamber. MFGE8-neutralizing antibody (720 ng/mL; 2422, MBL) or control hamster IgG (ebioscience) was treated for MFGE8 neutralization. Images were captured with an Eclipse Ti inverted microscope (Nikon, Tokyo, Japan).

### 2.8. Microarray and Data Analysis

Mo-MDSCs and Gr-MDSCs were treated with TGF-β during in vitro differentiation. After sorting, Mo- and Gr-MDSCs were lysed with TRI Reagent and extracted mRNA was analyzed on a GeneChip Mouse Gene 2.0 ST array (Affymetrix, Santa Clara, CA, USA). Three independent microarray experiments were performed. The resulting data was analyzed by Gene Set Enrichment Analysis (GSEA; https://www.gsea-msigdb.org/gsea/index.jsp, accessed on 9 March 2020) [34] and heat maps were generated using Multiple Experiment Viewer (MeV).

For the gene expression analysis of *Mfge8* in tumor-infiltrated MDSCs, the Gene Expression Omnibus (GEO) datasets GSE110772 and GSE21927 were retrieved and analyzed.

### 2.9. Statistical Analysis

The bar graphs show the means of 2–3 independent experiments and the error bars represent the standard deviation (SD). *p*-values were calculated using two-tailed Student’s *t*-tests, and *p*-values of <0.05 were considered statistically significant.

## 3. Results

### 3.1. TGF-β Is an Important Regulator of MDSCs Immunosuppressive Function

**Figure 1:**
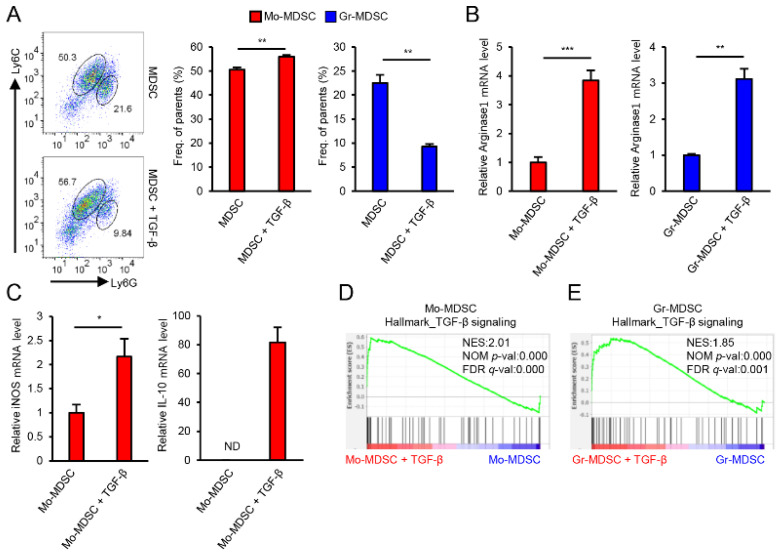
TGF-β increases the immunosuppressive function of MDSCs. (**A**) Representative FACS plots of MDSCs subpopulations [Mo-MDSCs (Ly6G^low^Ly6C^high^) and Gr-MDSCs (Ly6G^high^Ly6C^low^)] and bar graphs showing the percentages of each subpopulation. (**B**) The level of arginase1 mRNA in Mo-MDSCs and Gr-MDSCs after TGF-β treatment. (**C**) iNOS and IL-10 mRNA levels in Mo-MDSCs after TGF-β treatment under lipopolysaccharide (LPS) stimulation condition. (**D**) GSEA analysis of microarray data from Mo-MDSCs treated with TGF-β. (**E**) GSEA analysis of microarray data from Gr-MDSCs treated with TGF-β. Data are expressed as the mean ± SD. ND, not detected. * *p* < 0.05, ** *p* < 0.01, and *** *p* < 0.001 by two-tailed *t*-test.

Previously, we have shown that TGF-β is an important factor for the regulation of MDSCs’ immune suppressive function [7]. Therefore, in this study, to identify the differentially expressed genes related to the functional enhancement of MDSCs by TGF-β, MDSCs were treated with TGF-β and gene expression changes were analyzed by microarray. In this analysis, we confirmed that treatment with TGF-β increased the Mo-MDSC population and decreased the Gr-MDSC population, as shown in our previous report (Figure 1A). In addition, the MDSC effector molecules arginase1, iNOS, and IL-10 were up-regulated at the mRNA level by TGF-β treatment of MDSCs (Figure 1B,C). We also analyzed differential gene expression by GSEA and confirmed that TGF-β signaling pathway-related molecules were significantly enriched in both Mo-MDSCs and Gr-MDSCs treated with TGF-β (Figure 1D,E), indicating that the immunosuppressive function of MDSCs is increased by TGF-β.

### 3.2. TGF-β Induces Gene Expression Changes including Pro-Metastatic Genes Such as Mfge8

**Figure 2:**
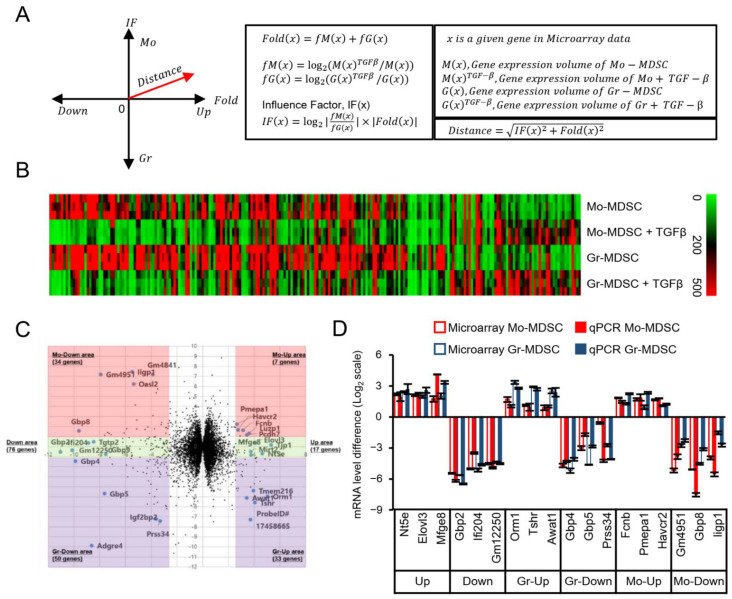
Gene expression profile in response to TGF-β treatment. (**A**) Graphical scheme and calculation scheme used in the two-dimensional plot. (**B**) Heat map of genes with a ‘Fold’ value > 2.5 in three independent microarray experiments. (**C**) Two-dimensional plot of genes from the microarray data. The *X*-axis represents ‘Fold’ and the *Y*-axis represents ‘IF’. (**D**) The top three genes in each of the groups were confirmed by qPCR. The changes in the mRNA levels upon TGF-β treatment by microarray or qPCR are shown. Data are expressed as the mean ± SD.

To analyze the TGF-β-induced differential gene expression in MDSCs, we created a two-dimensional plot of the microarray data (Figure 2A). The *X*-axis represents the Fold Change (‘Fold’), a sum of the gene expression changes in TGF-β-treated Mo-MDSCs and Gr-MDSCs on a log2 scale. The *Y*-axis represents the Influence Factor (‘IF’), the product of the ‘Fold’ and the difference between the fold changes in Mo-MDSCs and Gr-MDSCs on a log2 scale. Thus, the ‘IF’ represents whether TGF-β affected the expression of a given gene more strongly in Mo-MDSCs or Gr-MDSCs. ‘Distance’ is the distance from the origin of the plot; thus the ‘Distance’ is dependent on both the ‘Fold’ and the ‘IF’. From the microarray data analysis, 227 genes with a ‘Fold’ value greater than 2.5 were selected and displayed on a heat map (Figure 2B). The two-dimensional plot of the microarray data showed the differential gene expression profiles of Mo-MDSCs and Gr-MDSCs after TGF-β treatment (Figure 2C). The 227 genes were divided into six groups according to the ‘IF’ value, representing the differential impact of TGF-β on each subset of MDSCs. Genes with an ‘IF’ value greater than 1 (the impact of TGF-β on the expression of the gene was greater in Mo-MDSCs) were assigned to the ‘Mo-Up’ or ‘Mo-Down’ group. Genes with an ‘IF’ value lower than −1 (the impact of TGF-β on the expression of the gene was greater in Gr-MDSCs) were assigned to the ‘Gr-Up’ or ‘Gr-Down’ group. Genes with an ‘IF’ value greater than −1 and lower than 1 were assigned to the ‘Up’ or ‘Down’ group. Finally, the three genes with the largest ‘Distance’ in six groups were selected, and the TGF-β-induced differential mRNA expression was confirmed by qPCR (Figure 2D). 

TGF-β is a cytokine with various functions, including immune suppression [6,7] and tumor progression [23,24,35]. Therefore, we hypothesized that genes with differential expression by TGF-β treatment may have similar function with TGF-β and the 18 genes that were selected from the differential expression analysis described above were analyzed for their roles in inflammation or tumor progression (Table 1). Among the 18 genes, six genes (*Nt5e*, *Elovl3*, *Mfge8*, *Tshr*, *Gbp5*, and *Havcr2*) have been previously described to have roles in cancer progression or immune regulation. *Mfge8* was selected as a target gene of interest because *Mfge8* had the largest ‘Distance’ value when recalculated with the qPCR results (Table A1), and because *Mfge8* has an anti-inflammatory function [32,36] as well as a role in promoting tumor metastasis [28,30].

### 3.3. MFGE8 Does Not Promote the Immunosuppressive Function of MDSCs

**Figure 3:**
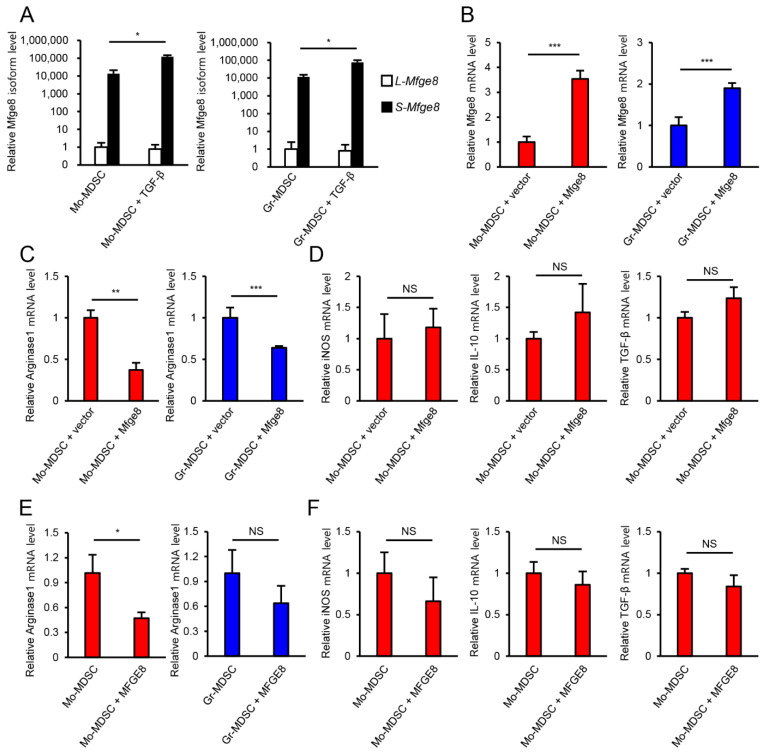
MFGE8 does not increase the immunosuppressive function of MDSCs. (**A**) Analysis of L-MFGE8 and S-MFGE8 mRNA levels in Mo-MDSCs and Gr-MDSCs by qPCR. (**B**) MFGE8 overexpression was confirmed by qPCR after sorting of transduced cells. (**C**) The arginase1 mRNA level in MFGE8-overexpressing Mo-MDSCs and Gr-MDSCs. (**D**) mRNA levels of MDSC effector molecules in MFGE8-overexpressing Mo-MDSCs under LPS stimulation condition. (**E**) The arginase1 mRNA level in MFGE8-treated Mo-MDSCs and Gr-MDSCs. (**F**) mRNA levels of MDSC effector molecules in MFGE8-treated Mo-MDSCs under LPS stimulation condition. Data are expressed as the mean ± SD. NS, not statistically significant (*p* > 0.05). * *p* < 0.05, ** *p* < 0.01, and *** *p* < 0.001 by two-tailed *t*-test.

As there are two isoforms of MFGE8, the short isoform (S-MFGE8) and the long isoform (L-MFGE8), we confirmed which isoform is expressed in MDSCs by qPCR before investigating the role of MFGE8 in MDSCs (Figure 3A). S-MFGE8 mRNA was preferentially expressed over L-MFGE8 in both Mo-MDSCs and Gr-MDSCs. Therefore, the following experiments were performed with S-MFGE8.

Next, we overexpressed S-MFGE8 in Mo-MDSCs and Gr-MDSCs using retroviral infection to find out whether MFGE8 promotes the immunosuppressive function of MDSCs. MFGE8 mRNA expression was increased in Mo-MDSCs and Gr-MDSCs upon infection with the MFGE8-expressing retrovirus compared with cells infected with the vector control retrovirus (Figure 3B). Next, the expression of MDSC immunosuppressive effector molecules was measured. Surprisingly, the arginase1 mRNA level decreased (Figure 3C) upon MFGE8 overexpression, and the levels of other effector molecules, including iNOS, IL-10, and TGF-β, were unaffected (Figure 3D). Although MFGE8 is known to have anti-inflammatory properties, it did not promote the expression of immunosuppressive effector molecules in MDSCs. MFGE8 treatment showed similar results to MFGE8 overexpression (Figure 3E,F). These results indicate that, in contrast to our expectations, MFGE8 did not promote the immunosuppressive function of MDSCs.

### 3.4. MFGE8 Induces Metastasis of B16F10 Melanoma

**Figure 4:**
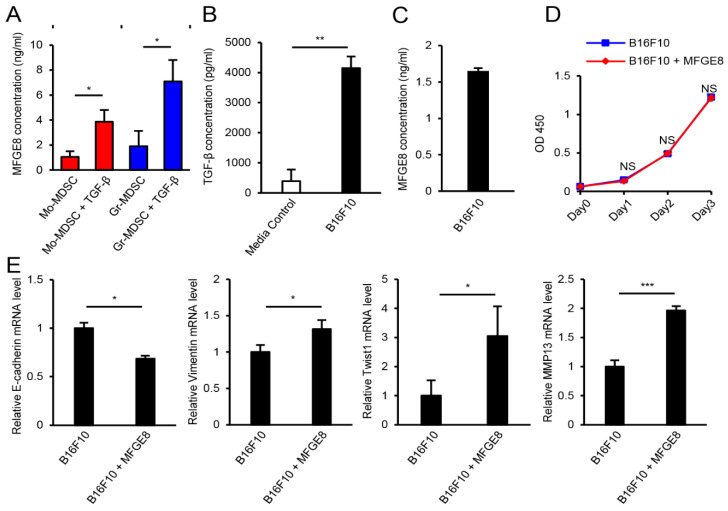
MFGE8 induces EMT of B16F10 melanoma. (**A**) ELISA of MFGE8 production by Mo-MDSCs and Gr-MDSCs in response to TGF-β treatment. (**B**) ELISA of TGF-β production by B16F10 melanoma cells. (**C**) ELISA of MFGE8 production by B16F10 melanoma cells. (**D**) WST assay of B16F10 melanoma cells in response to MFGE8 treatment. (**E**) mRNA levels of the EMT markers E-cadherin, vimentin, twist1, and MMP13 in B16F10 melanoma cells treated with MFGE8. Data are expressed as the mean ± SD. NS, not statistically significant (*p* > 0.05). * *p* < 0.05, ** *p* < 0.01, and *** *p* < 0.001 by two-tailed *t*-test.

**Figure A1:**
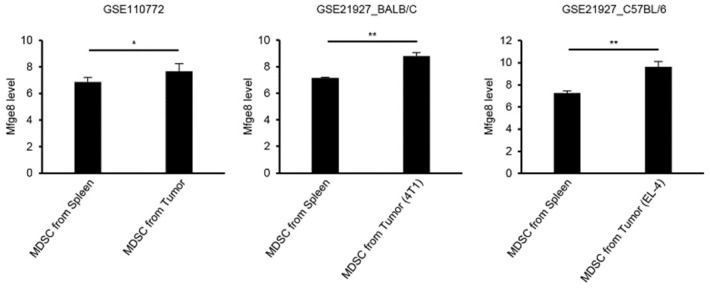
*Mfge8* expression is increased in tumor-infiltrated MDSCs. *Mfge8* expression in tumor-infiltrated MDSCs in the GEO datasets GSE110772 and GSE21927. Data are expressed as the mean ± SD. * *p* < 0.05 and ** *p* < 0.01 by two-tailed *t*-test.

**Figure A2:**
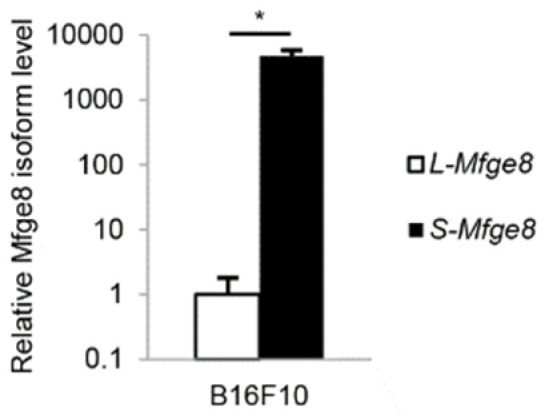
*S-Mfge8* expression level is higher than *L-Mfge8* in B16F10 cells. Analysis of L-MFGE8 and S-MFGE8 mRNA levels in B16F10 cells by qPCR. Data are expressed as the mean ± SD. * *p* < 0.05 by two-tailed *t*-test.

Although MFGE8 did not increase the immunosuppressive function of MDSCs, it has been previously reported that MFGE8 induces tumor metastasis in various models [27,30]. Therefore, we hypothesized that MFGE8, as an effector molecule of MDSCs, may promote cancer metastasis in TGF-β-rich environments such as the TME. To confirm MFGE8 secretion by MDSCs, we measured the MFGE8 concentration by ELISA (Figure 4A). Both Mo-MDSCs and Gr-MDSCs secreted MFGE8, and TGF-β significantly increased its secretion. Open-source microarray data showed that tumor-infiltrated MDSCs also express higher levels of MFGE8 mRNA than splenic MDSCs (Figure A1). Furthermore, to confirm B16F10 cells as a source of TGF-β, the concentration of TGF-β was measured by ELISA (Figure 4B). Since B16F10 cells can also secrete MFGE8, the concentration of MFGE8 protein was also measured in B16F10 supernatants by ELISA and isoform expression levels of MFGE8 in B16F10 were examined by qPCR (Figure 4C and Figure A2). B16F10 melanoma secreted MFGE8 (1.65 ± 0.04 ng/mL), which is similar amount of MFGE8 secreted by Mo-MDSCs and Gr-MDSCs without TGF-β (1.06 ± 0.44 ng/mL and 1.91 ± 1.23 ng/mL, respectively) where S-MFGE8 is preferentially expressed over L-MFGE8. However, both Mo-MDSCs and Gr-MDSCs secreted substantially higher amounts of MFGE8 in response to TGF-β treatment (3.86 ± 0.95 ng/mL and 7.10 ± 1.70 ng/mL, respectively). Therefore, these ELISA results indicate that MDSCs are the main source of MFGE8 in the TME. 

After confirming MDSCs and B16F10 as a source of MFGE8 and TGF-β, respectively, we evaluated B16F10 proliferation, EMT marker expression, and matrix metalloproteinase (MMP) expression after MFGE8 treatment. MMPs are enzymes that play an important role in cancer cell invasion. For tumor cells to become invasive, MMPs are required to degrade the basal membrane [53]. Although the proliferation of B16F10 cells was not affected by MFGE8 treatment (Figure 4D), MFGE8 induced EMT of B16F10 melanoma cells, as the expression of E-cadherin mRNA decreased while the expression of vimentin and twist1 mRNA increased. Moreover, the mRNA expression of MMP13 was significantly increased by MFGE8 treatment (Figure 4E). Taken together, these data show that MFGE8 induces metastasis of B16F10 melanoma. 

### 3.5. MDSCs Increase B16F10 Migration in an MFGE8-Dependent Manner

**Figure 5:**
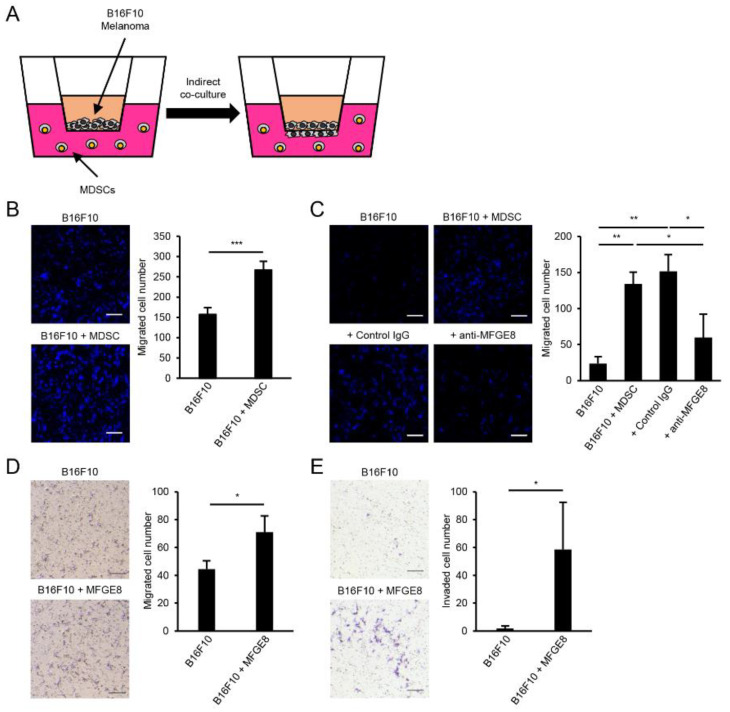
MFGE8 secreted by MDSCs affects B16F10 melanoma transmigration and invasion. (**A**) Schematic figure of co-culture experiments with MDSCs and B16F10 melanoma cells. (**B**) Co-culture of MDSCs and B16F10 melanoma cells. B16F10 cells were labeled with CellTrace Violet before co-culture. (**C**) Co-culture of MDSCs and B16F10 melanoma cells with MFGE8 neutralization. B16F10 cells were labeled with CellTrace Violet before co-culture. (**D**) Transmigration of B16F10 melanoma cells with MFGE8 treatment. (**E**) Invasion assay of B16F10 melanoma cells with MFGE8 treatment. Scale bars, 500 µm. Data are expressed as the mean ± SD. * *p* < 0.05, ** *p* < 0.01, and *** *p* < 0.001 by two-tailed *t*-test.

To further evaluate the relationship between MDSCs and B16F10 melanoma, we conducted a co-culture experiment of MDSCs and B16F10 cells (Figure 5A). Co-culture of B16F10 and MDSCs significantly increased B16F10 transmigration (Figure 5B). Furthermore, MFGE8 neutralization significantly reduced B16F10 transmigration (Figure 5C), while MFGE8 treatment increased B16F10 transmigration and invasion (Figure 5D,E). Since MFGE8 treatment did not affect B16F10 proliferation (Figure 4D), the increased cell numbers are a result of augmented B16F10 migration. These results suggest that MDSCs can induce B16F10 metastasis by secreting MFGE8 in response to TGF-β. These results highlight an interaction between MDSCs and B16F10 melanoma cells and demonstrate that MFGE8 is a key molecule that promotes B16F10 melanoma migration. 

## 4. Discussion

MDSCs accumulate and interact with tumor cells in the TME. It is widely accepted that MDSCs accumulation results in tumor progression and metastasis [54], however the detailed mechanisms are still not fully understood. In this study, we showed that MDSCs interact with B16F10 melanoma, increasing cancer cell migration. TGF-β treatment of MDSCs resulted in increased MFGE8 secretion and addition of MFGE8 neutralizing antibody demonstrated that MDSC-derived MFGE8 promoted the migration of B16F10 melanoma cells. Previous studies have shown that MFGE8 promotes tumor progression or metastasis in various cancer models [27,29,30], yet the reported source of MFGE8 was mainly TAMs or stromal cells. Therefore, our findings suggest a new mechanism through which MDSCs promote tumor progression, as a source of MFGE8. In addition, although MDSCs preferentially express S-MFGE8, comparing S-MFGE8 function with L-MFGE8 in the tumor microenvironment could be an important issue in future studies. 

In a previous study, we showed that TGF-β increased the immunosuppressive function of MDSCs in a murine colitis model [7]. However, the function of TGF-β is not restricted to prolonged inflammatory response alleviation. Many studies have shown the roles of TGF-β in tumor metastasis [24,25] and immune modulation. Here, we demonstrate an additional role of TGF-β as an immune modulator in the TME. TGF-β increased the Mo-MDSC population, which directly interact with tumor cells and induce EMT in the TME [19]. The immunosuppressive function of MDSCs was assessed based on their mRNA expression of effector molecules. Arginase1, iNOS, and IL-10 mRNA levels increased with TGF-β treatment. Most importantly, TGF-β altered the gene expression profile of MDSCs, upregulating pro-metastatic genes such as *Mfge8.* Although MFGE8 did not promote MDSCs immunosuppressive function itself, it affected the phenotype of B16F10 melanoma cells, suggesting a role of TGF-β and TGF-β—induced genes in tumor metastasis. Furthermore, MFGE8 produced by MDSCs may also be involved in the inhibition of T cell proliferation in the tumor microenvironment, as a previous report showed inhibition of T cell proliferation by MFGE8 [55]. 

Generally, cancer metastasis is related to the prognosis and survival rate. Often, melanoma can be detected easily, but it is highly metastatic, thus increasing the mortality rate [56,57]. In this study, we showed that MDSCs induce the migration of B16F10 mouse melanoma cells by secreting MFGE8, and MFGE8 neutralization suppressed their migration. A previous study also suggested MFGE8 antibody as a novel therapeutic for ovarian carcinoma [27]. Together, these results suggest the therapeutic potential of targeting MDSCs or MFGE8 for metastatic melanoma or other cancers. 

## 5. Conclusions

We demonstrate that TGF-β alters the gene expression profile of MDSCs. *Mfge8* and a number of other metastatic genes were upregulated in TGF-β-treated MDSCs. MFGE8 did not directly increase MDSC effector molecule expression, but MDSC-derived MFGE8 promoted the metastasis of B16F10 melanoma cells, suggesting that MDSC-derived MFGE8 could be a promising target for metastatic cancer therapies.

## Figures and Tables

**Table 1:** Known functions of the 18 genes selected from the microarray data. The top three genes in each of the six groups were selected for further analysis. The function of some genes has not been well-studied, and these genes are denoted as ‘unknown’.

Group	Gene Name	Function
Up	*Nt5e*	Promote tumor migration [37,38]
	*Elovl3*	Fatty acid chain elongation [39]
	*Mfge8*	Anti-inflammatory [40,41], Tumor metastasis [27,30]
Down	Gbp2 *	Better prognosis in breast cancer [42], Glioblastoma invasion [43]
	Ifi204 *	Negatively regulate type 1 interferon response [44]
	Gm12250 *	Unknown
Gr-Up	Orm1*	Therapeutic response predictor in NK/T lymphoma [45]
	*Tshr*	Inhibit metastasis of thyroid cancer [46]
	Awat1 *	Unknown
Gr-Down	Gbp4 *	Inflammasome activation [47]
	*Gbp5*	Inflammasome assembly [48], Stimulation of NF-κB signaling pathway [49]
	Prss34 *	Unknown
Mo-Up	Fcnb *	Maintain immunosuppressive function of Gr-MDSCs [50]
	Pmepa1 *	Prostate cancer metastasis regulator [51]
	*Havcr2*	T cell exhaustion and tolerance [52]
Mo-Down	Gm4951 *	Unknown
	Gbp8 *	Unknown
	Iigp1 *	Unknown

* Few studies done, or results are controversial.

## Data Availability

The datasets are available from the corresponding authors upon reasonable request.

## Appendix A

**Table A1:** ‘Distance’ values of six candidate genes. ‘Distance’ values of six genes were calculated based on the qPCR results.

Gene Name	Distance
*Nt5e*	5.13
*Elovl3*	4.97
*Mfge8*	7.78
*Tshr*	6.51
*Gbp5*	5.68
*Havcr2*	3.46
